# The Emerging Role of Mesenchymal Stem Cells in Vascular Calcification

**DOI:** 10.1155/2019/2875189

**Published:** 2019-04-01

**Authors:** Changming Xie, Liu Ouyang, Jie Chen, Huanji Zhang, Pei Luo, Jingfeng Wang, Hui Huang

**Affiliations:** ^1^Department of Cardiology, The Eighth Affiliated Hospital, Sun Yat-sen University, Shenzhen 518000, China; ^2^Guangdong Provincial Key Laboratory of Malignant Tumor Epigenetics and Gene Regulation, Department of Cardiology, Sun Yat-sen Memorial Hospital, Sun Yat-sen University, Guangzhou 510120, China; ^3^Department of Radiation Oncology, Sun Yat-sen Memorial Hospital, Sun Yat-sen University, Guangzhou 510120, China; ^4^State Key Laboratory for Quality Research of Chinese Medicines, Macau University of Science and Technology, Taipa 999078, Macau

## Abstract

Vascular calcification (VC), characterized by hydroxyapatite crystal depositing in the vessel wall, is a common pathological condition shared by many chronic diseases and an independent risk factor for cardiovascular events. Recently, VC is regarded as an active, dynamic cell-mediated process, during which calcifying cell transition is critical. Mesenchymal stem cells (MSCs), with a multidirectional differentiation ability and great potential for clinical application, play a duplex role in the VC process. MSCs facilitate VC mainly through osteogenic transformation and apoptosis. Meanwhile, several studies have reported the protective role of MSCs. Anti-inflammation, blockade of the BMP2 signal, downregulation of the Wnt signal, and antiapoptosis through paracrine signaling are possible mechanisms. This review displays the evidence both on the facilitating role and on the protective role of MSCs, then discusses the key factors determining this divergence.

## 1. Introduction

Vascular calcification (VC) is a pathological accumulation of calcium phosphate crystal depositing in the medial and intimal layers of the vessel wall. This common pathologic hallmark is shared by multiple chronic diseases. For example, atherosclerosis and its comorbidities, such as diabetes and chronic kidney disease (CKD), display this feature. Calcification is a major risk factor for cardiovascular mobility and mortality [1]. However, the exact mechanisms underlying VC are poorly characterized. Reliable clinical therapies are in high demand. However, there are no effective treatments currently able to reverse calcium deposition. Recently, VC is considered an active process regulated by cellular pathways resembling those participating in bone morphogenesis. Some cell types consisting of the arterial wall would reprogram their genetic expression patterns, transform into osteoblast-like cells, and initiate the mineralization of the extracellular matrix (ECM) in response to multiple stimulations, involving cyclic strain overload [2], inflammation [3], and metabolic disorder [4, 5]. The interaction between the bone and cardiovascular system gives rise to tremendous interest among researchers. For example, Cianciolo et al. and Fadini et al. found that bone marrow-derived cells could immigrate from circulation into vessels, transform into osteogenic cells, and then facilitate VC [6, 7]. One subpopulation of those bone marrow-derived cells is CD34+ (marker of hematopoietic stem cells) cells including endothelial precursor cells (EPCs) and calcifying myeloid cells. While the other is CD34- mesenchymal stem cells (MSCs) [6, 8].

Mesenchymal stem cells, also known as marrow stromal cells, bone marrow fibroblasts, or skeletal stem cells, are typically defined as follows: (1) MSCs must be plastic-adherent when maintained in standard culture conditions, (2) MSCs must express CD105, CD73, and CD90 and lack the expression of CD45, CD34, CD14 or CD11b, CD79*α* or CD19, and HLA-DR surface molecules, and (3) MSCs must differentiate into osteoblasts, adipocytes, and chondroblasts in vitro [9]. Based on their source and location, they could be classified as bone marrow- (BM-) MSCs, peripheral blood-MSCs, or pericytes [10]. As has been observed previously, MSCs are well demonstrated to exhibit remarkable immune regulation and anti-inflammation capacities such as angiogenesis in regenerative medicine [11]. Therefore, it is likely that MSCs are candidates to contribute to the alleviation of VC. According to Zhu et al., coculture of vascular smooth muscle cells (VSMCs) with BM-MSCs could inhibit vascular calcification via the Wnt signaling pathway [12]. However, Cho et al. calculated the calcium accumulation level of arteries in an atherosclerosis model and found it to be increased significantly after injecting MSCs [13].

For now, the role of MSCs in the VC process still remains unclear and controversial. Whether MSCs facilitate or inhibit VC is a pending question yet to be identified.

This review begins with a brief description of the physiological functions of MSCs and definition of VC, followed by a discussion of recent studies of MSCs in VC and their underlying pleiotropic mechanism.

## 2. Physiological Roles of MSCs in the Vascular System

Blood vessels are the most widely distributed tissue in the human body and are vital for the development, normal physiology, and most, if not all, human diseases. As one type of vascular progenitor cells, MSCs serve as essential participants in the formation, repair, and remodeling of arterial vessels [14]. It is widely accepted that MSCs could differentiate into endothelial cells, VSMCs, or pericytes [15–17]. Besides, MSCs have the capacity to promote angiogenesis by secreting proangiogenic factors or producing extracellular vesicles in a paracrine-dependent manner [18]. In addition, studies proved that MSCs are able to govern immunity and restrain inflammation. BM-MSCs could suppress T cell proliferation by secreting soluble factors with immunosuppressive activity, including indoleamine 2,3-dioxygenase (IDO), prostaglandin E2 (PGE2), interleukin 10 (IL-10), IL-6, and IL-17 [19]. All of these promising effects make them a potential therapy required for vascular repair and regeneration.

## 3. Characters of Vascular Calcification

VC refers to ectopic deposits of hydroxyapatite with a high degree of crystallization in the wall of vessels. VC frequently occurs in atherosclerosis, hypertension, diabetes, CKD, and aging [20]. Morphologically, VC can be divided into intimal and medial calcification. Calcification of the intimal layer usually occurs in large- and medium-sized elastic arteries [21]. It was considered a feature of advanced atherosclerosis and may be responsible for coronary ischemic events. However, some other research reported that most calcified plaques may be more stable and that the plaques that are most vulnerable to rupture may be those which have a mixed composition of calcified and noncalcified tissue [22]. Intimal VC is more relevant to vascular senescence and chronic inflammation [23]. Medial calcification, with pathological characteristics of nonocclusive and preferential development along elastic fibers, is dramatically increased in chronic kidney disease-mineral and bone disorder (CKD-MBD) [24]. Disturbances of calcium and phosphate metabolism, a perturbation of the bone vascular axis, and reduction of calcification inhibitors are all considered potential mechanisms.

Cells from all layers of the vessel wall could transform into osteoblast-like cells. Taking calcified VSMCs for example, they lost parts of their contractile phenotype, which is supported by downregulation of *α*-smooth muscle actin (*α*-SMA) and SM-22. Meanwhile, they are featured by abnormal increasing expression of the osteogenesis gene, for instance, Runt-related transcription factor 2 (Runx2), osterix, osteopontin (OPN), and osteocalcin (OCN) [3].

VC was initiated by matrix vesicles (MVs), which are produced by osteoblast-like cells and act as sites for hydroxyapatite crystal precipitation. Meanwhile, elastin is degraded due to the overexpression of matrix metalloproteinase by calcified cells, which in turn promotes VSMCs losing their contractile markers. Taken together, phenotypic transition is the driving factor during the calcification process.

## 4. Evidence of the Facilitating Role of MSCs in VC

As discussed above, osteoblast-like cells are the key contributor of VC. Owning the potential of osteogenic differentiation and recruitment to injury vessels, MSCs play a critical role in the “circulating calcifying cell theory”; in other words, they may act as a source of osteoblast-like cells.

Several in vitro studies provided direct evidences on the ability of MSCs to differentiate into osteoblast-like cells in VC cultured osteogenic media. When treated with dexamethasone, *β*-glycerophosphate (*β*-GP), and L-ascorbic acid, murine MSCs can be induced to osteoblast-like cells that have strong expression of type I collagen and bone morphogenetic protein-2 (BMP2) and are positive in von Kossa staining [25]. Uremic serum can induce a calcific phenotype in human MSCs in a BMP2/4-dependent manner, accompanied by matrix remodeling and calcification [26], which may serve as a mechanism underlining CKD-related bone disorder. Moreover, MSCs isolated from ApoE-/- mice showed a significant increase in in vitro osteogenesis and chondrogenesis in a cartilage intermediate, which indicates that MSCs may contribute to the ectopic calcification of atherosclerotic plaque [27].

Circulating MSCs can migrate through the blood stream and reach the site of injury in the vessel wall. VC often emerges as a secondary alteration of vessel damage, where a similar recruitment process can be found. Several chemokines have been reported to be involved in the MSC recruitment process of VC: the accelerating effect of transforming growth factor (TGF-*β*) in VC has been widely accepted [28–30]. Intravenous injection of recombinant active TGF-*β*1 in uninjured mice rapidly mobilized MSCs into circulation with an amplification effect by the cascade expression of monocyte chemotactic protein-1 (MCP-1) [31, 32]. In a model of crossing LDLR-/- mice with transgenic mice, fed with high-fat western diets, in which all the MSC-derived cells were fluorescently labeled, Wang et al. reported that both active TGF-*β*1 mouse levels and MSCs in circulating blood were upregulated at the same time points when these cells appeared at the aortic tissue and lately VC appeared severely. Immunohistochemistry staining showed that the increased active TGF-*β*1 level was seen throughout the whole wall of the aorta. [30]. As a potent mitogen and chemoattractant, a platelet-derived growth factor (PDGF) has been found to disturb the vascular homeostasis by inflammation, oxidative stress, and phenotype transition, all of which accelerate the process of VC [8]. PDGF-BB was found to be most effective in stimulating MSC migration among other PDGF isoforms and even TGF-*β*, BMP2, and SDF-*α* [33]. Interestingly, in Fiedler et al.'s research about vascular calcifying progenitor cells, the chemotactic effect of PDGF-AB exceeds that of PDGF-BB in the case of primary osteoblasts, which reveals a subtype specificity. Under some pathological conditions such as renal ischemia-reperfusion injury and inflammatory cardiomyopathy, stromal cell-derived factor 1 (SDF-1) promotes homing of MSCs to injury sites and enhances the retention of infused cells [34–37]. Wu et al. demonstrated that sympathetic denervation could increase bone formation in distraction osteogenesis. Norepinephrine promotes in local vessels the secretion of SDF-1, which attracts MSCs staying in vessels instead of migrating to the lesioned bone [38]. Parathyroid hormone (PTH), which is unregulated pathologically in CKD, induces an increased expression of SDF-1 through the downregulation of dipeptidylpeptidase IV [39]. Here, we summarize the potential chemokines of MSCs in Table 1.

More directly, Cho et al. calculated the calcium accumulation level of arteries in an atherosclerosis model and found it to be increased significantly after injecting calcifying progenitor cells [13]. Transplantation of BM-MSCs induced vascular remodeling and calcification after balloon angioplasty in hyperlipidemia rats [40]. Another previous study showed increased intramyocardial calcification that resulted from MSC homing after direct transplantation of unselected BM cells [41]. In heterotypical transplantation of MSCs with an established three-dimensional collagen-based skeleton to rat models of CKD, aortas and MSC-containing collagen gels showed distinct similarities in the calcification and upregulation of the osteolytic markers and ECM remodeling with increased expression of osteopontin, collagen I/III/IV, fibronectin, and laminin [42]. To assess the intrinsic calcification capacity of MSCs and the effect of the atherosclerotic environment, a similar experiment where MSCs loaded on collagen-glycosaminoglycan scaffolds were implanted subcutaneously to ApoE-/- was conducted [27]. From above, it is disappointing to find that VC can be a potential side effect of MSC therapy. However, more frustratingly, MSCs are also reported to be involved in VC under many pathological stages in vivo. A research described the biological behavior of adventitial Gli1+ MSCs in ApoE-/- mice with CKD: MSCs migrated into the media in both CKD and sham groups. During 16 weeks after nephrectomy, where severe calcification occurred, they differentiate into VSMCs firstly but eventually lost the expression of VSMC markers and turn to osteoblast-like cells that have strong costaining for Runx2 and are located within calcium tracer-positive areas. This research proposed that MSCs are a major source of osteoblast-like cells during VC [43]. Chlamydia pneumoniae infection may promote VC by indirectly stimulating the phenotypic conversion of MSCs [44].

## 5. Evidence of the Protective Role of MSCs in VC

MSCs have been identified as an effective agent for application in various diseases/complications including VC. Wang et al. have proved that the bioactive substance secreted by MSCs could retard murine VSMC calcification induced by *β*-GP with conditioned medium from MSCs (MSC-CM) [45, 46]. Consistent with the above study, Zhu and her colleagues established the indirect coculture system of VSMCs and MSCs with Transwell. Calcification of VSMCs in the lower layer with osteogenic medium was significantly decreased [12].

According to previous evidence, there are four potential pathways involved in MSCs protection of VC.

### 5.1. Inhibition of Inflammation

The MSC-CM is well known to be a rich source of autologous cytokines, based on which cell-free stem treatment was developed. Various factors derived from MSC-CM such as IL-4, IL-6, and IL-1RA are capable of expressing an anti-inflammatory effect [47, 48], which have been proven to play a role in lung injury, myocardial infarction, and corneal wound [46]. For myocardial infarction, a novel research reports that MSC-derived exosomes can improve the microenvironment contributing to angiogenesis and anti-inflammation [49]. The close association of VC with inflammation has been summarized by many excellent papers [50–52]. Directly, TNF-*α*, IL-1*β*, and IL-6, which play crucial roles in the initiation and progression of VC, were found to be suppressed when treated with MSC-CM [45]. NF-*κ*B is a crucial pathway in vascular inflammation [53]. It was downregulated when MSCs' paracrine function was enhanced in lipopolysaccharide-induced inflammation.

### 5.2. Blockade of the BMP2-Smad1/5/8 Signaling Pathway

Wang et al. firstly demonstrated that MSC-CM suppression of calcification may be mediated by the expression of bone morphogenetic protein-2 (BMP2) and the BMP2 receptor-Smad1/5/8 signaling pathway [46]. BMPs are a superfamily of transforming growth factor-beta (TGF-*β*) and secretory growth factor, which play a role in bone formation. As described above, BMPs are reported to be expressed strongly in VC and accelerated atherosclerotic intimal calcification in BMP2 transgenic/ApoE-knockout mice [25, 26, 54]. Unfortunately, how MSCs suppress the BMP2 signal is still unclear.

### 5.3. Downregulation of the Canonical Wnt Signaling Pathways

Three Wnt pathways have been described: the Wnt/*β*-catenin (canonical pathway) [55], the Wnt/Ca2+ noncanonical pathway, and the noncanonical planar cell polarity pathway (PCP), all of which have been implicated in human cardiovascular diseases [56]. The crucial role of Wnt signaling pathways in VC has already been proven by a large number of researches [57, 58], which will be further discussed in the following part. In the indirect coculture study, the activities of canonical and noncanonical Wnt ligands (Wnt5a), receptor tyrosine kinase-like orphan receptor 2 (Ror2), and *β*-catenin were downregulated [12]. Similarly, how this suppression works remains unknown.

### 5.4. Inhibition of Apoptosis

Cell apoptosis is regulated by the expression of caspase-3 and the ratio of the antiapoptotic factor Bcl-2 to the proapoptotic factor Bax. This ratio of VSMCs is rescued by MSC-CM in a *β*-GP-induced VC model [45]. Apoptotic bodies of VSMCs have the capacity to concentrate and crystallize calcium to initiate VC [59]. More directly, bone-targeted overexpression of Bcl-2 in mouse osteoblasts suppressed calcification in vitro [60].

To sum up, the protective role of MSCs in VC is mainly in a paracrine-dependent manner. Autologous cytokines secreted by MSCs regulate VSMC biological behavior in the process of VC. However, further exploration is needed.

## 6. Possible Mechanisms Determine the Role of Mesenchymal Stem Cells in VC

MSCs differentiate to osteoblast-like cells then promote VC. However, they lead the protective role in a paracrine manner. That is a brief summary of the role of MSCs in VC. However, it is not clear enough what determines the ultimate effect of VC. The potential factors will be discussed as follows.

### 6.1. The Microenvironment of the Vessel

Early on, scientists learned the importance of the microenvironment (also called niche) in the fate of stem cells, in both retaining stemness and differentiation. Plenty of studies indicate that differentiated cells could influence MSC differentiation. Direct coculture of MSCs with endothelial cells (ECs) resulted in an increase in *α*-smooth muscle actin mRNA and protein of MSCs but also a comprehensive disruption of *α*-smooth muscle actin filament organization [61]. For VC, cell components change a lot. Take VC in atherosclerosis as an example, there are many pathological differentiated cell types, such as foam cells, osteogenic phenotype VSMCs, and ECs with a decrease in physiological vessel cells. Using an in vitro cell-cell coculturing system, Xin et al. observed that MSCs directly interact with normal or calcified VSMCs. Osteosynthesis-inducing medium (OS) treatment did not promote the generation of an osteoblast phenotype in cultured MSCs. However, MSCs exhibited an osteoblast phenotype when MSCs were cocultured in direct contact with calcified VSMCs whether with or without OS treatment [62]. That is completely opposite to the aforementioned indirect coculture research [12].

Regrettably, this study did not give us a contact-related explanation. Instead, the results are reported in a Wnt signaling-dependent manner. LRP5, a receptor of the canonical Wnt pathway, was upregulated, while Ror2, the receptor of the noncanonical pathway, was downregulated in MSCs [62, 63]. Canonical Wnt/*β*-catenin signaling is a significant pathway in VC. In phosphorus-induced calcification, this signal was upregulated [58]. Another in vitro model of human VSMC calcification was induced by exposure to high glucose. The Wnt signaling molecules including Wnt3a, Wnt7a, and Fzd4 were highly expressed, and the phosphorylation of *β*-catenin was increased, which can be inhibited by Dkk1, a Wnt signaling inhibitor [57]. As for the downstream genes, many osteogenesis genes, such as osteocalcin type I collagen, Runx2, osteopontin, and autophagy, upregulate type III Na-Pi cotransporters (PiT1), and lymphoid enhancer-binding factor (LEF) has been proven to be the target genes [64–67]. Actually, the Wnt signal was reported earlier in MSC osteogenic transformation in physiological bone and cartilage formation [68–70]. It is not surprising to find a similar effect in VC. When the canonical Wnt signal is suppressed in MSCs by sFRP2, interestingly, MSCs' self-renewal capacity is enhanced, which promotes engraftment and myocardial repair [71]. Taken together, inappropriate activation of the Wnt signal in the microenvironment may result in both VSMC and MSC osteogenesis transformations to facilitate VC.

More recently, one study provides comprehensive evidence that osteoblast-derived small extracellular vesicles in the culture environment were of critical importance. The extracellular vesicles were successfully applied to induce BM-MSC differentiation towards a mineral phenotype [72].

**Figure 1:**
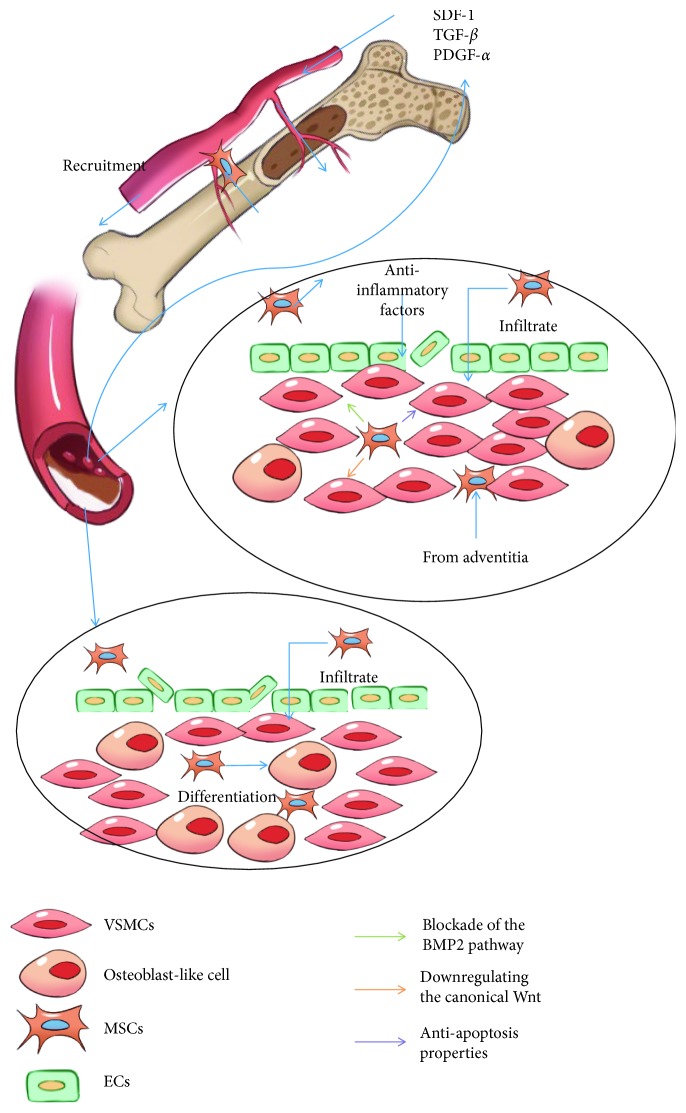
A brief illustration of MSCs and VC and alternation of the microenvironment. In the damaged vessel under the calcification process, SDF-1, PDGF, and TGF-*β* are released to recruit MSCs from bone marrow and circulation. (a) In this microenvironment, damage of the vessel wall is slight and the effect of oxidative stress and inflammation is very minimal. In addition, fewer VSMCs have been induced to osteoblasts. MSCs are viable and inhibit VSMC osteogenesis differentiation by a paracrine mechanism. (b) In this microenvironment, the vessel is damaged a lot by heavy oxidative stress and inflammation. Several phenotypic transformations of VSMCs have taken place. MSCs tend to undergo apoptosis and differentiate into osteoblast-like cells, which facilitate the VC progress.

Going through the researches associated with the protective role of MSCs, we found that they all kept the MSCs away from the calcified vascular microenvironment. That means that MSCs can exert the protective effect only when they maintain the ability of stemness. Neither MSC transplantation therapy nor endogenous recruitment can avoid MSCs being affected by the pathological condition. As a result, they facilitate VC (see Figure 1).

### 6.2. Low Survival Rate of MSCs

It has been reported that less than 1% MSCs survive for more than one week after systemic administration [73, 74], which is a huge challenge in stem cell therapy. The reasons are complicated, one of which is the overload of oxidant stresses. Environmental stress induces excessive production of reactive oxygen species (ROS), which are capable of initiating oxidation and causing a variety of cellular responses, such as DNA damage [75]. Oxidant pressure from hyperlipidemia is a potential common etiology of VC, atherosclerosis, and osteoporosis [76]. After being recruited, MSCs will be continuously exposed to oxidants under the pathological microenvironment and induced to necrosis and apoptosis [77, 78].

However, the story for facilitating VC is quite different. Dead MSCs still have a residual effect to promote VC. Recently, exosomes secreted by osteoblasts or osteoblast-like cells, which are characterized by decreased calcifying inhibitors and increased phosphatidylserine and annexin A6 content, can initiate calcification by acting as crystallization cores [79, 80] and their capacity to concentrate and crystallize calcium as well [59]. That was partly confirmed by Fujita and his collages: apoptosis and necrosis occurred in an osteogenic culture of MSCs and cell death preceded calcification. Spontaneously dead cells by osteogenic culture and exogenously added necrotic cells were surrounded by calcium deposits [81]. Besides, antioxidants (tiron and N-acetylcysteine) inhibited cell death and calcification. This could be partly confirmed by ineffective efferocytosis, which is the main mechanism operating in fibroatheroma [21]. The accumulation of apoptotic bodies established a vicious circle with inflammatory response. However, it has been described that under an in vitro osteogenic microenvironment, MSCs derived from the human arterial wall are able to release exosomes with high affinity for hydroxyapatite crystal, which indicates that viable MSCs facilitate VC as well [21].

**Figure 2:**
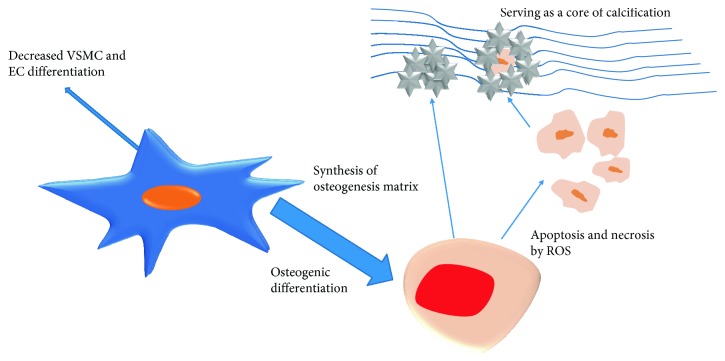
How the MSCs propel the calcification process: in the calcification microenvironment, MSCs are induced to differentiate into osteoblast-like cells, which synthesize the osteogenesis matrix. On the other hand, under adverse condition, MSC apoptosis or necrosis happens. Then, the fragments serve as a core of calcification deposit. Meanwhile, less MSCs differentiate into VSMCs and ECs, which creates a vicious cycle.

Compared with an in vitro experiment, an in vivo model preferably mimics the in-suit environment which is generally harder for MSCs to survive [73, 74]. The residual effect of dead MSCs may partly account for the procalcification effect found in vivo. However, further studies are still needed (see Figure 2).

## 7. Conclusions

Over the years, it is apparent that VC occurs in a wide range of vascular pathologies and is a tightly regulated process. MSCs, a natural “repairman” and promising stem cell therapy agent, may lose part of their beneficial effects and promote VC [7]. MSCs facilitate VC mainly through osteogenesis differentiation. Even necrotic or apoptotic MSCs have the capacity to concentrate and crystallize calcium as well. However, the protective role only acts through a paracrine mechanism which required high cell vitality. The mechanism remains rarely known. The crosstalk between MSCs and inflammatory mediators has been proven to determine the procalcific remodeling of human atherosclerotic aneurysm [50]. However, not only inflammation but also the alternation of the microenvironment is a driving factor, which impacts the differentiation fate and function of MSCs. The survival rate of MSCs is a huge challenge that not only limits the beneficial effect but also enhances the membrane fraction of necrotic cells and apoptotic bodies. With more clues being discovered, the role of MSCs in VC progression is increasingly clear, which is helpful to illuminate the underlying mechanism of VC.

## Figures and Tables

**Table 1:** Chemokines of MSCs.

Factors	Main characters	Reference
TGF-*β*	(1) TGF-*β* can be released by the damaged vessel cells and lesioned artery and involved in vascular regeneration and VC (2) TGF-*β* couples bone resorption with formation by inducing MSC migration and participates in bone and cartilage metabolism. Subchondral bone MSCs activated by TGF-*β* seem to initiate osteoarthritis (3) TGF-*β* promotes homing of BM-MSC in a tissue lesion, for example, renal ischemia-reperfusion injury (4) TGF-*β* may regulate the SDF-1/CXCR4 axis and MCP-1 to induce MSC homing	[30–32, 36, 82–87]

PDGF-BB	(1) PDGF has the highest effect among other cytokines (SDF-1a, CXCL16, MIP, etc.), and PDGF-BB is the most strong one among PDGF isoforms in vitro (2) PDGF-BB has been proven to be involved in myocardial and lung functional tissue regeneration, angiogenesis, and VC by recruiting MSCs (3) PDGF-BB has been applied for bone regeneration and proven to recruit MSCs to the scaffolds	[13, 33, 82, 88–92]

PDGF-AA	(1) PDGF-AA's chemotaxis effect is lower than that of PDGF-BB, but stronger in recruiting osteogenic differentiated progenitor cells (2) PDGF-AA can promote MSC proliferation and differentiation (3) The effect of PDGF-AA can be blocked by TGF-*β*	[82, 93–95]

SDF-1	(1) SDF-1 can be released by the endothelium and ischemic myocardium in myocardial infarction, inflammatory cardiomyopathy, and vascular injury. This cytokine also correlated with the severity of calcification (2) In inflammatory bone destruction, SDF-1 was found to be upregulated, which could possibly enhance fracture healing in osteoporotic patients by recruiting MSCs. And it also improves the vascularization of bone (3) SDF-1 promotes MSCs to repair liver injury, expanded skin, and even cancer (4) Serum SDF-1 can be increased by hypoxemia	[34, 35, 37, 88, 96–104]

BMP2/4/7	It has only been proven in vitro	[82, 89]

FGF	In vivo researches of FGF chemotaxis mainly focus on pulmonary fibrosis	[82, 88, 105, 106]

VEGF	(1) Chemotactic activity of VEGF has been proven in vitro. And VEGF can be released by multiple myeloma and glioma cells to improve vascularization (2) VEGF plays a role in bone regeneration (3) PDGFR-*α* is required	[82, 89, 107–110]

G-CSF	(1) In vivo chemotactic activity of G-CSF is controversial (2) It may work via CXCR4/SDF-1	[100, 111–113]

TNF-*α*/IL-1*β*/IL-6	These cytokines are associated with inflammation and work through the NF-*κ*b pathway. And several researches show that they inhibit instead of promoting migration	[88, 114–118]

IGF-1	Chemotactic activity of IGF-1 is not so assuring. Pretreatment seems more reliable	[89, 119–122]

PTH	PTH can improve osteoporosis in mice and men and spine injuries	[39, 123]

The table shows chemokines of MSCs with a brief introduction of their characters. TGF: transforming growth factor; PDGF: platelet-derived growth factor; SDF: stromal cell-derived factor; BMP: bone morphogenetic protein; FGF: fibroblast growth factor; VEGF: vascular endothelial growth factor; G-CSF: granulocyte colony-stimulating factor; TNF: tumor necrosis factor; IL: interleukin; IGF: insulin-like growth factor; PTH: parathyroid hormone.
